# The Diagnostic Performance of Serum Glycosylated Ferritin in Patients Undergoing Regular Blood Transfusion: An Indicator of Iron Overload to Initiate Iron Chelation Therapy

**DOI:** 10.7759/cureus.66695

**Published:** 2024-08-12

**Authors:** Aadarsh Shrivastava, Suchitra Kumari, Sonali Mohapatra

**Affiliations:** 1 Biochemistry, All India Institute of Medical Sciences, Bhubaneswar, Bhubaneswar, IND; 2 Hematology, All India Institute of Medical Sciences, Bhubaneswar, Bhubaneswar, IND

**Keywords:** blood transfusion, iron overload, transferrin saturation, glycosylated ferritin, ferritin

## Abstract

Background and objective

Serum ferritin concentration and transferrin saturation are commonly employed to estimate body iron but are non-specific to iron overload. Glycosylated ferritin may be primarily elevated in cases of iron overload in patients undergoing regular blood transfusions. In this study, we aimed to estimate glycosylated ferritin and determine its cutoff values for iron overload in patients receiving blood transfusions regularly. We also endeavored to the examine correlation between serum ferritin and glycosylated ferritin in patients receiving regular blood transfusions.

Methods

We conducted a cross-sectional study involving 17 patients undergoing regular blood transfusions in the Department of Medical Oncology/Hematology, who had already received ≥10 transfusions without any iron chelation therapy or acute inflammation. All participants were evaluated based on a questionnaire to gather relevant medical details. Serum iron, ferritin, glycosylated ferritin, and unsaturated iron-binding capacity (UIBC) were estimated. Total iron-binding capacity (TIBC) and transferrin saturation were also calculated.

Results

Participants were divided into two groups based on transferrin saturation (≥50% as a reference for iron overload). The group with transferrin saturation ≥50% had significantly higher levels of serum ferritin, glycosylated ferritin, and iron, compared to the group with transferrin saturation <50%. Glycosylated ferritin showed a positive correlation with ferritin (rho=0.80) and transferrin saturation (rho=0.64), which was statistically significant. UIBC and TIBC showed a negative association with glycosylated ferritin. The correlation of glycosylated ferritin with units of blood transfusion (Spearman’s rho=0.60) was found to be better than that of serum ferritin (Spearman’s rho=0.52).

Conclusions

Based on our findings, glycosylated ferritin could be a potential marker for transfusion-related iron overload. The optimal cutoff value for iron overload using serum glycosylated ferritin level was >587.55 ng/mL. Further extensive studies with larger sample sizes will substantiate the role of glycosylated ferritin in predicting post-transfusion iron overload.

## Introduction

Repeated blood transfusions are frequently required in patients with chronic hematological diseases such as β thalassemia, sickle cell anemia, aplastic anemia, and myelodysplastic syndromes (MDS) [1]. Post-transfusion iron overload is one of the major concerns in these patients, as excess iron may accumulate in vital organs (heart, liver, etc.) in the form of ferritin or hemosiderin, which can have severe, life-threatening consequences [1,2]. Therefore, the estimation of body iron becomes vital in patients undergoing regular blood transfusions. The ways of estimation include direct methods - liver and/or heart biopsy - and indirect methods - ferritin concentration, transferrin saturation, heart and liver MRI, and superconducting quantum interface device (SQUID) [3,4].

For patients with iron overload induced by recurrent red blood cell (RBC) transfusions, iron chelation therapy is employed to improve survival and quality of life. Currently, serum ferritin levels greater than 1000 ng/mL and a liver iron concentration (LIC) greater than 3 mg iron/g dry weight, as measured by liver biopsy or hepatic T2* MRI, are the indications to start iron chelation therapy [5]. While non-invasive methods such as biomagnetic liver susceptibility (BLS) and MRI are very accurate [4,6], due to their limited availability and very high cost, they are seldom used in our clinical setting. Serum ferritin levels could be a valuable screening approach, but it lacks specificity as its levels are elevated in conditions such as liver failure, cancers, and inflammatory and infectious diseases, thereby limiting its use as a measure of iron overload [3,7]. In light of these findings, it is necessary to identify an effective and clinically useful biomarker for determining transfusion-related iron overload status.

Ferritin is present in two forms: glycosylated and non-glycosylated [8]. Around 50-80% of serum ferritin is said to be glycosylated [9]. Patients with adult-onset Still’s disease, an illness traditionally linked with hemophagocytic syndrome, have high total serum ferritin (~3344 ng/mL) with a relatively low percentage of glycosylated ferritin (20%), indicating that the non-glycosylated part of ferritin increases in inflammatory conditions. Its utility as a biomarker of an inflammatory condition is being studied [8,10,11]. Glycosylation seems to be partly mediated by reticuloendothelial or parenchymal cells. A recent study showed glycosylated ferritin as an improved marker for post-transfusion iron overload [12]. However, the correlation between the values of glycosylated ferritin and iron overload status has not been established. Also, there are no defined cutoff levels for glycosylated serum ferritin for initiating iron chelation therapy. This study was conducted to estimate glycosylated ferritin and determine its cutoff values for iron overload in patients receiving blood transfusions regularly. Furthermore, the correlation between serum ferritin and glycosylated ferritin in patients receiving regular blood transfusions was evaluated.

## Materials and methods

Study design and setup

This was a cross-sectional study conducted in the Department of Biochemistry, All India Institute of Medical Sciences, Bhubaneswar from July 2022 to September 2022. It involved 17 patients undergoing regular blood transfusion as a day-care treatment in the Department of Medical Oncology/Hematology who have already received transfusions ≥10 times. Patients in whom iron chelation therapy had already been initiated, along with those experiencing acute inflammation and infectious diseases, were excluded from this study. The sample size was calculated to be 28 based on the correlation data provided by a previous study by Ishihara et al. [12] with an alpha error of 5% and a power of 80%. However, only 17 patients were recruited due to time constraints, a limited number of patients, and narrow inclusion criteria.

All participants were evaluated based on a questionnaire related to the study, which included confirmed diagnosis, age at diagnosis, frequency of blood transfusion, amount of blood transfused in every session, number of units of packed red blood cells (1 unit=350 mL) transfused to date, iron chelation therapy status, signs of acute inflammation, and history of recent infectious diseases.

After obtaining informed written consent, 3 mL of blood samples were collected from all participants and processed to separate the serum. The serum was stored at -20 °C until it was used for biochemical evaluation. Values of serum iron and total iron-binding capacity (TIBC) were measured by a fully automated Chemistry analyzer (Beckman Coulter AU5800, Brea, CA). Transferrin saturation was calculated using the following formula:

$\text{Transferrin Saturation (\%)} = \left( \frac{\text{Serum Iron}}{\text{Total Iron-Binding Capacity}} \right) \times 100$

Serum ferritin was estimated by a one-step direct immunoassay using chemiluminescent technology with the chemiluminescence immunoassay analyzer. Glycosylated ferritin was measured using an ELISA kit (Cat ELK 8575, ELK Biotechnology, Wuhan, China). The clinical findings as well as biochemical reports were recorded in the case study form for the patients.

Patients were segregated according to their iron status based on serum transferrin saturation, with a value ≥50% treated as iron overload.

Statistical analysis

The data were tabulated using MS Excel 2019, and data analysis was conducted using IBM SPSS Statistics v26 (IBM Corp., Armonk, NY). The normality of the data was assessed, and descriptive statistics were reported as mean ± standard deviation (SD) or median [interquartile range (IQR)] accordingly. Correlation between various parameters was assessed using Pearson’s r or Spearman’s rho. ROC analysis was performed to analyze diagnostic indices. A p-value <0.05 was considered statistically significant.

## Results

The clinico-demographic details of the study participants are presented in Table 1. Both male and female patients aged between 1 and 23 years were enrolled in this study. Participants were classified into two groups based on transferrin saturation. The group with transferrin saturation ≥50% exhibited significantly higher levels of total serum ferritin, glycosylated ferritin, and serum iron compared to the group with transferrin saturation <50%.

**Table 1:** Descriptive statistics and comparison between groups based on transferrin saturation ^*^Mann-Whitney U test (p-value <0.05 is considered significant) BMI: Body mass index. IQR: Interquartile range. TIBC: Total iron-binding capacity. UIBC: Unsaturated iron-binding capacity

Parameter		Overall	Transferrin saturation <50%	Transferrin saturation ≥50%	P-value^*^
		n=17	n=10	n=7	
Sex	Male, n	10	6	4	-
	Male, n%	58.82	60	57.14	
	Female, n	7	4	3	
	Female. n%	41.12	40	42.86	
Age, years	Median	13	14	12	0.088
	IQR (Q1, Q3)	10, 15	12.25, 16	3.75, 13.5	
Blood transfused, units	Median	14	10	15	0.962
	IQR (Q1, Q3)	10, 18	4.75, 13	18, 19	
Height, m	Median	1.49	1.495	1.2	0.07
	IQR (Q1, Q3)	1.2, 1.5	1.47, 1.55	0.8, 1.5	
Weight, kg	Median	34.6	38.5	20	0.019
	IQR (Q1, Q3)	20, 42	34.6, 47	9.4, 33	
BMI, kg/m^2^	Median	16.27	18.01	14.67	0.055
	IQR (Q1, Q3)	13.89, 18.47	14.98, 20.72	13.75, 16.28	
Total serum ferritin, ng/mL	Median	980	730	2148	<0.001
	IQR (Q1, Q3)	700, 1690	468.2, 856	1680, 3876	
Glycosylated serum ferritin, ng/mL	Median	558.08	317.45	659.43	0.005
	IQR (Q1, Q3)	142.26, 656.53	87.98, 558.08	571.28, 1107.58	
Serum iron, μg/dL	Median	120.3	96.4	176.2	<0.001
	IQR (Q1, Q3)	84, 166.2	73, 114.7	131.9, 215.2	
UIBC, μg/dL	Median	145.9	204.45	85.1	<0.001
	IQR (Q1, Q3)	104, 213.8	151.5, 260.1	27, 116.3	
TIBC, μg/dL	Median	273.8	309	269	0.133
	IQR (Q1, Q3)	251.9, 325.5	254.7, 351.8	205.4, 282.5	
Transferrin saturation, %	Median	47.12	32.46	67.42	
	IQR (Q1, Q3)	30.61, 58.83	19.04, 42.72	58.57, 86.71	

As shown in Table 2, a significant positive correlation was observed between serum ferritin levels and glycosylated ferritin levels (Spearman’s rho=0.80), serum iron (Spearman’s rho=0.67), and transferrin saturation (Spearman’s rho=0.83). Unsaturated iron-binding capacity (UIBC) and total iron-binding capacity (TIBC) exhibited a negative association with serum ferritin levels. Glycosylated ferritin showed a positive correlation with serum ferritin (rho=0.80) and transferrin saturation (rho=0.64), both of which were statistically significant.

**Table 2:** Correlation of total serum ferritin and glycosylated ferritin with other parameters ^*^Spearman’s rho. ^**^P-value <0.05 is considered statistically significant TIBC: Total iron-binding capacity. UIBC: Unsaturated iron-binding capacity

Parameter	Ferritin		Glycosylated ferritin	
	Correlation coefficient^*^	P-value^**^	Correlation coefficient^*^	P-value^**^
Total serum ferritin, ng/mL	-	-	0.800	<0.001
Glycosylated serum ferritin, ng/mL	0.800	<0.001	-	-
Serum iron, μg/dL	0.679	0.004	0.460	0.063
UIBC, μg/dL	-0.909	<0.001	-0.748	<0.001
TIBC, μg/dL	-0.544	0.026	-0.548	0.023
Transferrin saturation, %	0.836	<0.001	0.641	<0.001
Blood transfused, units	0.521	0.032	0.606	0.010

**Figure 1:**
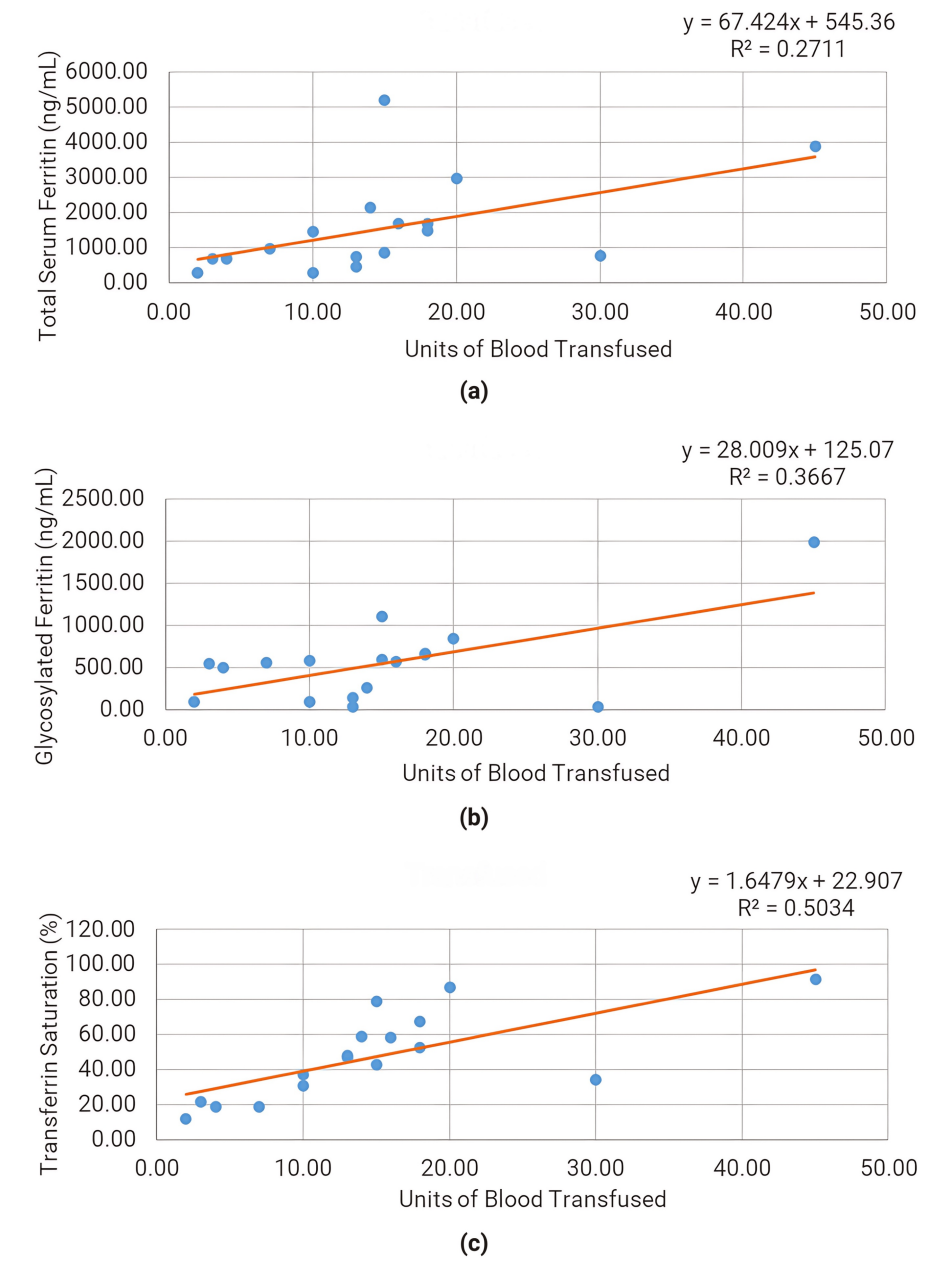
Scatter plot for various iron profile parameters with units of blood transfused (a) Scatter plot for total serum ferritin with units of blood transfused. (b) Scatter plot for glycosylated ferritin with units of blood transfused. (c) Scatter plot for transferrin saturation with units of blood transfused

The correlation between glycosylated ferritin and units of blood transfusion (Spearman’s rho=0.60) was found to be stronger than that between serum ferritin and units of blood transfusion (Spearman’s rho=0.52), as illustrated in Figure 1.

**Figure 2:**
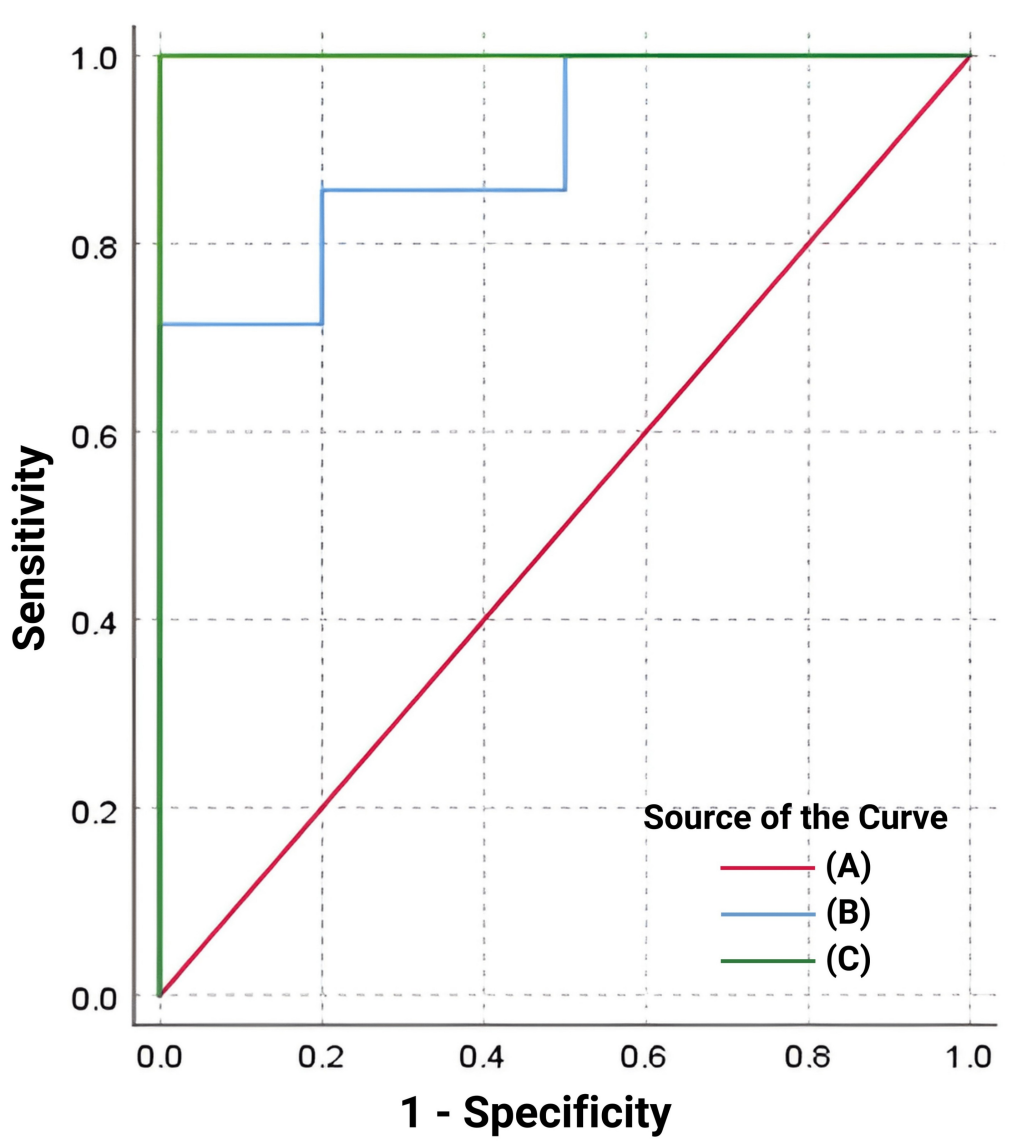
ROC (receiver operating characteristic) analysis of total serum ferritin and glycosylated ferritin for iron overload (A) Reference line. (B) Glycosylated serum ferritin (ng/mL). (C) Total serum ferritin (ng/mL)

To evaluate the role of serum ferritin and glycosylated ferritin as indicators of iron overload, with transferrin saturation ≥50% as a reference, a receiver-operating characteristic (ROC) analysis was performed. The area under the curve was 1.0 (95% CI: 0.805-1.000; p=0.001) for serum ferritin and 0.90 (95% CI: 0.658-0.991, p<0.001) for glycosylated ferritin, as depicted in Figure 2. The optimum cutoff value for diagnosing iron overload using serum ferritin level was >1457.0 ng/mL, with 100% sensitivity and 100% specificity. For serum glycosylated ferritin level, the best cutoff value for diagnosing iron overload was >587.55 ng/mL, with 71% sensitivity and 100% specificity, as shown in Table 3.

**Table 3:** Area under the curve (AUC) and diagnostic indices comparison for total serum ferritin and glycosylated ferritin ^*^P-value <0.05 is considered statistically significant

Indices	Total serum ferritin	Glycosylated ferritin
AUC (95% CI, p-value^*^)	1.000 (0.805–1.000, <0.001)	0.900 (0.658–0.991, <0.001)
Cutoff value (Youden Index)	>1457.0 (1.00)	>587.55 (0.71)
Sensitivity	100.00	71.43
Specificity	100.00	100.00

## Discussion

Frequent RBC transfusions often lead to iron overload since iron is minimally excreted from the body. Iron deposition in organs such as the liver, heart, and pancreas can cause free radical-mediated organ damage through reactions like the Haber-Weiss and Fenton reaction [13]. Iron chelation therapy is crucial in preventing such organ damage, thereby improving survival and quality of life in patients receiving repeated RBC transfusions. Serum ferritin plays a vital role in quantifying iron overload, and according to recent guidelines, ferritin levels should be measured at one to three monthly intervals [14]. However, ferritin, being an acute-phase reactant, might get elevated following tissue damage and inflammation. Serum ascorbate levels also affect ferritin levels.

While tissue iron quantification by biopsy and various MRI approaches are considered gold standards, they are not clinically practiced due to their high costs and limited availability in peripheral setups. TIBC, calculated indirectly through the summation of serum iron and UIBC, reflects the excess amount of iron needed to fully saturate transferrin. It serves as a measure of total serum transferrin concentration since most plasma iron is bound to transferrin. Hawkins et al. have found transferrin or TIBC measurements to be better indicators than serum iron levels in predicting iron deficiency, as iron levels exhibit circadian variation [15]. Recently, transferrin saturation has come to be regarded as an indicator of iron overload, with thresholds suggested by the European Association for the Study of the Liver (EASL) guidelines [16].

In the present study, significantly elevated levels of serum ferritin, glycosylated ferritin, and serum iron were observed in patients with transferrin saturation ≥50% compared to those with transferrin saturation <50%, consistent with earlier studies [16,17]. Transferrin saturation positively correlated with the units of blood transfusion, suggesting its role as an indicator of iron overload. Daru et al. [18] found serum ferritin to be a routinely available and well-correlated indicator of iron status, while Dignass et al. [19] discussed many limitations of ferritin as a marker of iron overload, especially in conditions of inflammation. In the present study, glycosylated ferritin correlated positively with serum ferritin (rho=0.80) and transferrin saturation (rho=0.64), which was statistically significant.

The correlation of glycosylated ferritin with the units of blood transfusion was found to be better than that of serum ferritin. Ferritin inside cells remains non-glycosylated, whereas in serum, ferritin is 60-80% glycosylated. This glycosylation occurs when ferritin is secreted from tissue into the serum [8], and glycosylation appears to be partly mediated by reticuloendothelial or parenchymal cells. Serum ferritin concentration increases in response to inflammatory disorders, infection, or malignant diseases, but in such situations, the glycosylated proportion of serum ferritin remains unchanged as ferritin synthesis is augmented inside cells in response to such scenarios, but not glycosylation.

Based on ROC analysis, we found that the area under the curve was 1.0 (95% CI: 0.805-1.000; p=0.001) for serum ferritin and 0.90 (95% CI: 0.658-0.991, p<0.001) for glycosylated ferritin, suggesting that glycosylated ferritin also has comparable efficacy to serum ferritin in indicating iron overload. The best cutoff value for diagnosing iron overload using serum ferritin level was >1457.0 ng/mL, with 100% sensitivity and 100% specificity. For serum glycosylated ferritin level, the best cutoff value for diagnosing iron overload was >587.55 ng/mL, with 71% sensitivity and 100% specificity.

Glycosylation of ferritin modifies certain properties of the circulating protein and influences the rate of removal; the half-life of glycosylated ferritin is approximately 50 hours compared to approximately five hours for non-glycosylated ferritin [10]. This difference could be responsible for the better correlation of glycosylated ferritin with the amount of RBC units transfused, irrespective of inflammatory status. In the present study, glycosylated ferritin was found to be primarily elevated in iron overload associated with RBC transfusions and showed a better correlation with the amount of RBC units transfused, irrespective of inflammatory status.

This study has a few limitations, primarily its small sample size and large variability, which was due to the narrow inclusion criteria and the short timeframe in which the study was conducted. A few strengths of this study include the fact that it is a pilot study to establish the role of glycosylated ferritin, and it is the first study to be conducted among our population.

## Conclusions

Our findings showed that glycosylated ferritin could serve as a potential marker for transfusion-related iron overload. The best cutoff value for iron overload using serum glycosylated ferritin level was >587.55 ng/mL. The correlation of glycosylated ferritin with units of blood transfusion was found to be better than that of serum ferritin. Further extensive studies with a larger and more homogeneous sample will help substantiate the role of glycosylated ferritin as an indicator of post-transfusion iron overload.
